# Analysis of PubMed User Sessions Using a Full-Day PubMed Query Log: A Comparison of Experienced and Nonexperienced PubMed Users

**DOI:** 10.2196/medinform.3740

**Published:** 2015-07-02

**Authors:** Illhoi Yoo, Abu Saleh Mohammad Mosa

**Affiliations:** ^1^ Department of Health Management and Informatics School of Medicine University of Missouri Columbia, MO United States; ^2^ Informatics Institute University of Missouri Columbia, MO United States; ^3^ Institute for Clinical and Translational Science School of Medicine University of Missouri Columbia, MO United States

**Keywords:** PubMed, MEDLINE, information retrieval, experienced users, nonexperienced users, PubMed query log

## Abstract

**Background:**

PubMed is the largest biomedical bibliographic information source on the Internet. PubMed has been considered one of the most important and reliable sources of up-to-date health care evidence. Previous studies examined the effects of domain expertise/knowledge on search performance using PubMed. However, very little is known about PubMed users’ knowledge of information retrieval (IR) functions and their usage in query formulation.

**Objective:**

The purpose of this study was to shed light on how experienced/nonexperienced PubMed users perform their search queries by analyzing a full-day query log. Our hypotheses were that (1) experienced PubMed users who use system functions quickly retrieve relevant documents and (2) nonexperienced PubMed users who do not use them have longer search sessions than experienced users.

**Methods:**

To test these hypotheses, we analyzed PubMed query log data containing nearly 3 million queries. User sessions were divided into two categories: experienced and nonexperienced. We compared experienced and nonexperienced users per number of sessions, and experienced and nonexperienced user sessions per session length, with a focus on how fast they completed their sessions.

**Results:**

To test our hypotheses, we measured how successful information retrieval was (at retrieving relevant documents), represented as the decrease rates of experienced and nonexperienced users from a session length of 1 to 2, 3, 4, and 5. The decrease rate (from a session length of 1 to 2) of the experienced users was significantly larger than that of the nonexperienced groups.

**Conclusions:**

Experienced PubMed users retrieve relevant documents more quickly than nonexperienced PubMed users in terms of session length.

##  Introduction

### Background

Methods of information seeking have become much easier, faster, and inexpensive since the 1990s with the advent of information technologies (ITs) including the Internet, digital libraries (eg, electronic full-text databases), and online search software/services such as Google Scholar and PubMed. [1-3]. Since then, immense change in scientific-information-seeking behavior has been observed, including among professionals, scholars, and scientists in the area of biomedical and health sciences [3-6]. There is unprecedented growth of biomedical information, which has been doubling every 5 years [7,8]. This large amount of scientific information from multiple sources (eg, journals) is currently integrated in electronic bibliographic databases and accessible through online search software [3,9]. For example, PubMed, which is maintained by the United States National Library of Medicine (NLM), is one of the largest and most authoritative online biomedical bibliographic databases in the world [10-12]. As of June 2015, PubMed contained more than 24 million citations and abstracts from approximately 5600 biomedicine and health-related journals. Health care professionals consider PubMed to be one of the most important and reliable sources of up-to-date health care evidence [13,14]. PubMed also plays a very important role in the process of literature-based discovery [15].

Recent years have seen a rising trend in biomedical information seeking from PubMed [16,17]. About two-thirds of PubMed users are domain experts (eg, health care professionals) and one-third are lay people [18]. Previous studies have examined the effects of domain expertise/knowledge on search performance using PubMed [6,19-21]. However, very little is known about PubMed users’ knowledge of information retrieval (IR) functions and their usage in query formulation.

The goal of this study was to shed light on how PubMed users perform their search queries by analyzing a full-day query log. The hypotheses of this study were that (1) experienced PubMed users who use system functions such as Medical Subject Heading (MeSH) terms and search field tags quickly retrieve relevant documents and (2) nonexperienced PubMed users who do not use them have longer search sessions than experienced users, because they identify their information needs through subsequent queries by narrowing and/or broadening their queries. In order to test the hypotheses, we analyzed a full day of PubMed log data. We assumed that if a session was closed within a few queries, the session was successful (meaning that relevant documents were retrieved), even if a session close did not always mean successful IR.

In this study, experienced PubMed users were defined as users who used advanced PubMed IR functions for query formulation. The proper use of IR functions (described in the next section) is key for efficient and effective PubMed searches [6,8,22-27] because, unlike Google, PubMed does not sort search results by relevance. Studies have shown that experienced users are more likely to use IR functions than novice users. Xie and Joo (2012) [28] performed a study on factors affecting the selection of search tactics and demonstrated that expert participants were more willing to use advanced IR functions. The study [28] used the definition of expert IR users from Holscher and Strube (2000) [29], in which expert users were defined as users having the “knowledge and skills” necessary to utilize information-seeking systems successfully. Holscher and Strube (2000) [29] also recognized that “expert users use advanced IR functions much more than average users.” Earlier studies also demonstrated that experienced searchers are more knowledgeable of the content and structure of the IR system and more likely to interact with the system [30,31]. Penniman (1981) [32] defined experienced PubMed users based on the frequency of PubMed searches and concluded that experienced searchers use more search functions than nonexperienced searchers. In addition, many studies have demonstrated that experienced users use more advanced IR functions and show better IR performance than novices [33-37].

### PubMed System Functions

PubMed system functions include search field tags, MeSH terms (used for indexing PubMed articles), truncation, and combining searches using search history. In PubMed, bibliographic information is stored in a structured database with 65 fields including title, abstract, author, journal or proceeding, publication type, and publication date. PubMed provides 48 search field tags in order to facilitate searching in its various database fields; a description for each search field is available at the NLM website [38] (last revised and updated November 2012). Thus, PubMed is a field-oriented search system in which search terms are tagged with search field tags and appended using Boolean operators (ie, AND, OR, and NOT). Using search field tags, PubMed users can limit the search to a specific field for each search keyword. A search field tag is attached to a search term by enclosing the search field name in square brackets (eg, "myocardial infarction" [Title]). The NLM indexes PubMed documents using the MeSH vocabulary after indexers read full papers (not just abstracts). Usually, 5-10 MeSH terms are assigned to a PubMed document. Truncation is used to search for the first 600 variations of a truncated word in PubMed. However, PubMed allows an asterisk (*) at the end of a word only; “?” is not used in PubMed. For example, the search term nutrition* will search for nutritional and nutritionists. Finally, the combining search function using search history enables PubMed users to readily use and combine previous search results using Boolean operators and search history indexes. For example, after a PubMed search for *diabetes mellitus*, the search result can be readily combined with one using a new search keyword *hypertension*: #1 AND *hypertension* (#1 indicates *diabetes mellitus*).

### Related Studies

The study of information-seeking behavior is very important for the user-centric design of online IR systems including digital libraries. Individuals’ knowledge and skills related to information seeking are the primary determinants of their online IR performance. According to Marchionini (1995) [39], there are four types of expertise that determine information-seeking performance: general cognitive abilities, domain knowledge, overall experience of online information seeking, and experience or knowledge of the functions of the IR system. Most intellectual activities like the information-seeking process involve planning (eg, query term selection), progress monitoring (eg, the number of returned documents), decision making (eg, when to continue or stop the search), and reflecting on past activities (eg, refining the search query for a better search result). Marchionini (1995) [39] stated that people’s perceptual and cognitive processes (known as cognitive abilities) are used in completing these tasks. As a common expectation, a person with higher cognitive abilities should perform better at information seeking than someone with lower cognitive abilities. However, few studies have investigated which cognitive abilities are linked to information seeking performance [1,29,39-42]. Hersh et al (2002) [24] assessed three cognitive factors (spatial visualization, logical reasoning, and verbal reasoning) that were found to affect IR performance, and found that PubMed/MEDLINE search experience and spatial visualization were the main factors in successful PubMed searches.

The second major area of expertise is the knowledge of information seekers in their area of interest (known as domain knowledge). The NLM reported that almost two-thirds of PubMed users are health care professionals and scientists (ie, domain experts), whereas the remainder are the general public [18]. Studies have demonstrated that methods of conducting information seeking tasks by domain experts are different from those of novice users [1,5]. In addition, overall IR performance of domain experts is better than that of novice users in various IR systems such as web and hypertext searches [29,42-46], and online bibliographic database searches [33,42,47]. A similar result has also been observed for PubMed searches [20,48]. PubMed search studies demonstrated that PubMed users with domain knowledge usually spent less time and retrieved more information than PubMed users with less domain knowledge. On the other hand, some studies measured user-searching performance (in terms of recall and precision) and concluded that domain knowledge did not significantly affect information-seeking performance. These studies were performed with the DIALOG database [49], an online library catalog [50], and the MEDLINE search system [19-21].

The other two determinants of search performance (ie, overall experience using online information seeking and experience or knowledge of the functions of the IR system) can be considered together as procedural knowledge for using the IR system [6]. Previous studies have demonstrated that such experience improves IR performance for various search systems such as web, hypertexts, file collections, and bibliographic DBs including PubMed [21,24,35,42,44,45,51]. Egan (1988) [52], Hölscher and Strube (2000) [29], and Jenkins et al (2003) [44] found that domain knowledge helped to improve search performance only if users had sufficient procedural knowledge including experience with online searching and search software/systems. In their literature review, Vibert et al (2009) [6] mainly compared the effects of domain knowledge on PubMed searches between expert and novice groups, and demonstrated that domain knowledge does not help to improve search performance if users do not have procedural knowledge. In addition, the study [6] suggested that knowledge in a broad scientific field can compensate for a lack of knowledge in a specific domain, and that the main determinant of bibliographic search performance is individual cognitive abilities. Thus, people with basic domain knowledge in their area of interest, higher cognitive abilities, and sufficient procedural knowledge regarding the bibliographic search system should efficiently perform information-seeking tasks (eg, query selection and decisions about search discontinuation). Some recent studies found that most academic researchers and health care professionals including physicians do not use advanced IR functions but only natural language for PubMed searches [6,51,53-55]. Another very recent study of PubMed by Macedo-Route et al (2012) [56] concluded that the way researchers use PubMed is nearly the same as the way IR novices do (“mostly typing a few keywords and scanning the titles retrieved by the tool”). Several studies have shown that medical librarians (considered experienced users in the study) use more IR functions for PubMed searches and their IR performance is better than regular users [20,36,57,58].

In this study, our goal was to compare experienced versus nonexperienced users’ searching behavior in terms of session length (ie, the number of queries per session). We used a full-day PubMed query log for that purpose. There are a number of approaches for studying user-searching behavior such as eye tracking, surveys, and search log analysis. Search log analysis has become a viable solution for many applications including search engines [16,17,59-63]. One major advantage of search log analysis over other methods is that actual searches by a large number of real users can be analyzed, while other methods usually examine searches from only tens up to hundreds of users. A search engine stores users’ query text along with other information including user IP addresses in query log files.

Silverstein et al (1999) [59] and Jansen et al (2000) [60] analyzed a query log from the AltaVista and Excite web search engines, respectively. Silverstein et al (1999) [59] reported three important facts: (1) users rarely navigate beyond the first page of search results, (2) they rarely resubmit a refined query (similar to Jansen et al (2000)’s [60] finding), and (3) most queries are short in length. Herskovic et al (2007) [16] carried out a similar study with a PubMed log and reported statistical information on PubMed usage (including the number of users, queries per user, sessions per user, and frequently used search terms and search field tags). The PubMed log data were used for segmenting query sessions [64], evaluating the PubMed Automatic Term Mapping (ATM) [65], and annotating PubMed queries using the Unified Medical Language System (UMLS) [66]. NLM researchers used month-long PubMed log data for categorizing PubMed queries [17,66], creating a query suggestion database [67], and identifying related journals for user queries [68]. Both of the full-day-long and month-long datasets are publicly available. However, the month-long dataset does not contain actual user queries. For this reason, we used the full-day-long PubMed log data.

The focus of this study is different from that of the eight studies that used PubMed log data [16,17,63-68]. We focused on comparing experienced versus nonexperienced users’ searching behavior in terms of session length (the number of queries in a session). To the best of our knowledge, there is no study with this focus.

## Methods

### Data Cleaning and Preprocessing

The dataset used in this study is a plain text file containing a full-day’s query log of PubMed that was obtained from the NLM FTP site (Refer to [69] to access the data). There are nearly 3 million queries issued by 626,554 distinct users.

**Figure 1:**
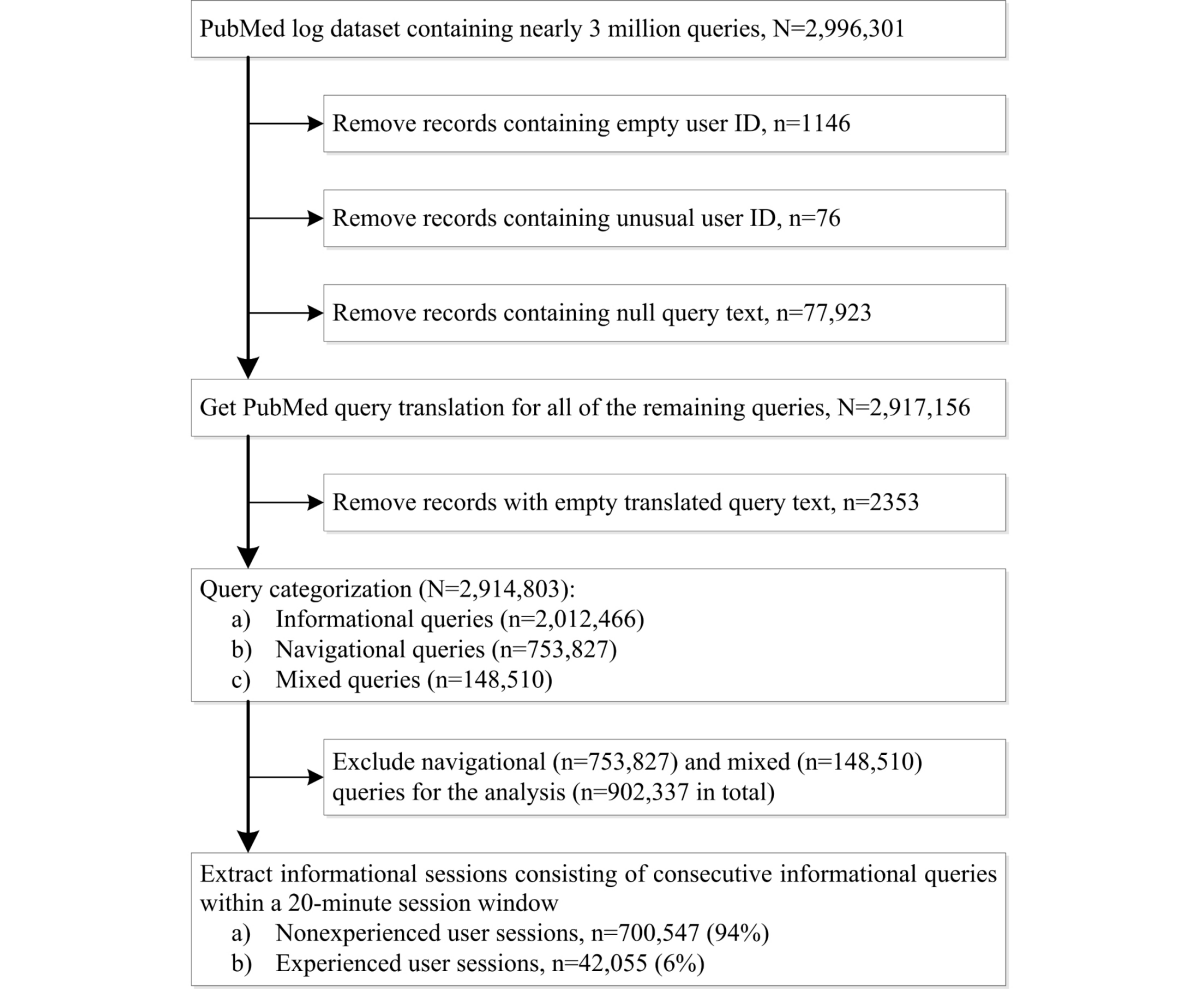
Data cleaning and preprocessing.

The data cleaning and preprocessing steps are presented in Figure 1. We found 1146 records with empty user IDs, 76 records with unusual user IDs (we believe they were errors), and 77,923 records with no user-query text. These records (79,145/2,996,301, 2.64%) were eliminated from the dataset.

### Query Categorization

The user queries in the PubMed log file are categorized as informational, navigational, or mixed according to the purpose of the search expressed in the query. Informational queries are intended to fulfill end users’ information needs (eg, "diabetes mellitus" [MeSH]) and navigational queries are intended to retrieve specific documents (eg, Yoo [author] AND Mosa [author]). Mixed queries have both intentions (eg, searching for a specific topic within a specific journal). Refer to Broder (2002) [70] and Herskovic et al (2007) [16] for details of web search types and PubMed search types, respectively.

**Figure 2:**
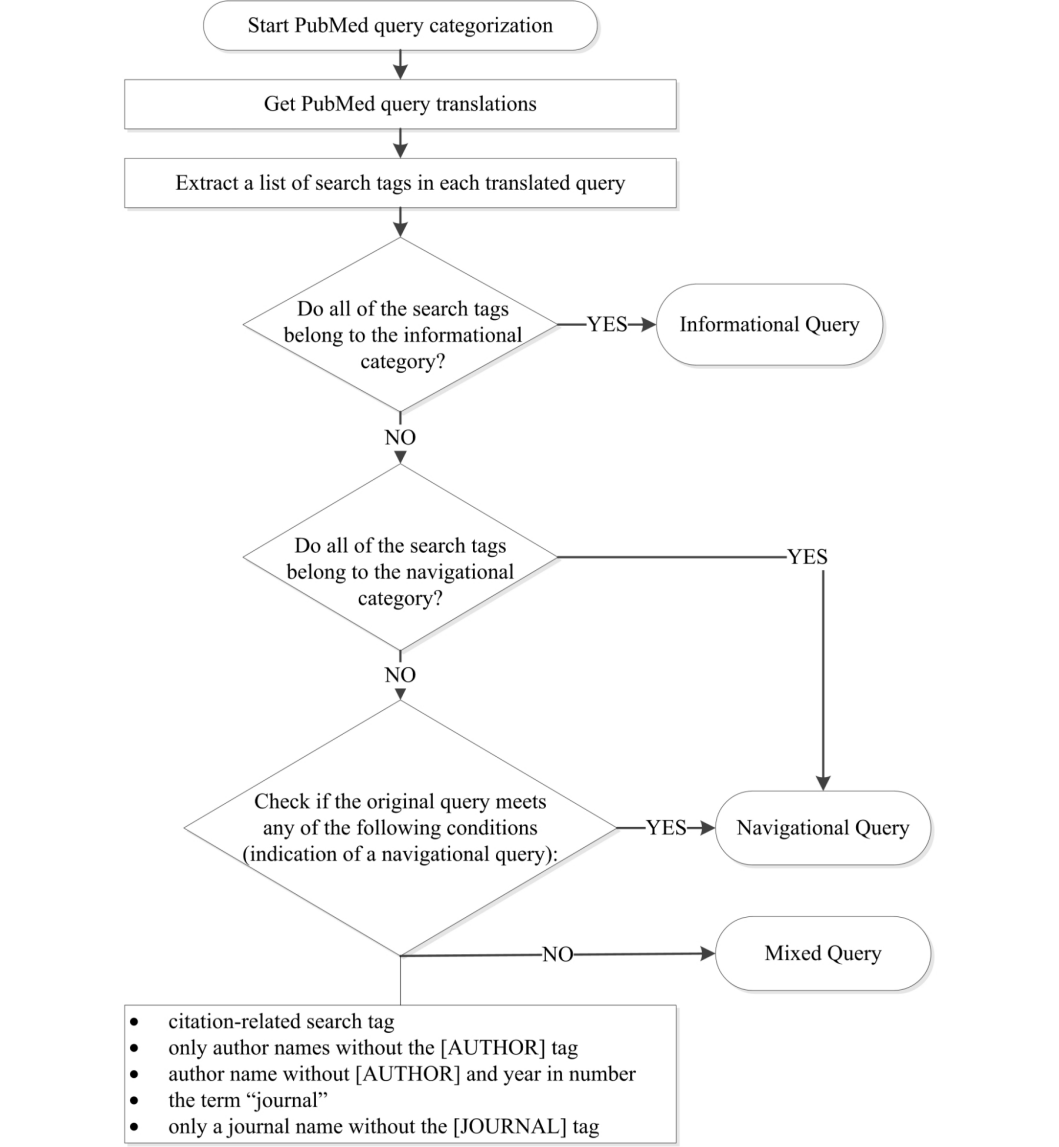
Query categorization.

In order to identify the purpose of user queries for query categorization, we used PubMed’s ATM. Every PubMed user query is automatically translated by ATM to improve overall IR performance and the translated query is actually used for the PubMed search; if a query contains double quotation marks or search tags, those parts (words or terms) are not translated. The ATM translation identifies each term in a query and adds an appropriate search tag to the term. We categorized PubMed queries using ATM-added tags as well as user-added tags after ATM translations. PubMed provides 48 search tags (refer to the PubMed Help website [71] for details), which are classified into informational and navigational tags [69]. Queries containing only informational tags are identified as informational queries. Navigational queries are queries containing navigational or citation-related tags. Queries containing both informational and navigational tags are identified as mixed queries, unless the original query contains an indication of a navigational query. Figure 2 presents a flow diagram for query categorization. A total of 2353 queries resulted in empty query translation. These were removed from the analysis. The translated query texts were then parsed to extract the search tags.

The search tag extraction process involved a semiautomatic approach consisting of two steps: the semiautomatic construction of a list of search tags and their variations, and the automatic extraction of the search tags including their variations from the queries using the search tag list. A total of 963 unique substrings were extracted from the queries in the first step. The first step (a partial manual step) was required for two reasons: (1) for each search tag there are several variations that are not fully documented even though they are correctly recognized by the PubMed system; for example, [Author Name], [Author], [AU Name], [Auth], and [AU] represent the same search tag header but only [Author Name] and [AU] are documented in the PubMed Help web page, and (2) incorrect search tags (eg, typos like [Atuhor]) used in PubMed queries are not recognized by the PubMed system but a domain expert could correctly recognize and read those intentions. The extracted search tags from the translated queries were then analyzed to identify query types. Since navigational search tags are mainly used to retrieve specific documents rather than to fulfill information needs, we excluded navigational and mixed queries from the analysis, assuming informational search tags are primarily used for information needs.

### Session Segmentation

Information seeking is defined as “the process of repeatedly searching over time in relation to a specific, but possibly an evolving information problem” [72]. Swanson et al (1977) [73] defined information seeking as a trial-and-error process, in which the initial search query is refined at every step, based on the search results in the previous queries. IR users often perform multiple queries in a row for the same information problem. The IR community has coined the term session in this regard. Silverstein et al (1999) [59] defined a session as “a series of queries by a single user made within a small range of time; a session is meant to capture a single user’s attempt to fill a single information need.” In order to segment queries by a user into sessions, most studies utilized temporal clues such as temporal threshold (ie, time cutoff) between two consecutive queries [59,74-78] or temporal constraint [79] (Refer to a recent survey article by Gayo-Avello (2009) [80] for details). This process (ie, session segmentation) provides valuable insights into users’ search behavior and interactions with the IR system.

In this study, we employed both the session-shift and temporal-constraint-based sliding window for session segmentation. This is because several studies reported the average duration of user sessions for query log analysis (meaning that the maximum length of session window can be chosen based on those results for session segmentation) [81-83]. In our study, we set the maximum length of the sliding window to be 20 minutes. The choice of a 20-minute session window was based on two biomedical IR studies. The first was a qualitative study with human subjects that showed most PubMed users successfully completed their task within a 15-minute period, whereas many took more than 15 minutes [6]. The second was a randomized controlled trial on biomedical information retrieval demonstrating that the average time to solve a biomedical information problem ranges from 14 to 17 minutes [84]. In addition to temporal constraint, we used change of query types as session shift. As a result, a change from an informational query to a navigational query was considered a session boundary.

Using this method, we extracted 742,602 user sessions from more than 2 million informational queries. User sessions were divided into two categories: experienced and nonexperienced. Experienced sessions were those in which queries were formed using system functions such as MeSH terms and search field tags. Otherwise, a user session was considered nonexperienced. For example, while a query containing “hypertension [MeSH]” was considered experienced, a query with “high blood pressure” was considered nonexperienced, even though hypertension is a synonym of high blood pressure. This is because although for the query “high blood pressure,” PubMed’s ATM internally expands the query by adding the MeSH term *hypertension*, the MeSH term is ORed with the term high blood pressure (i.e., hypertension [MeSH] OR high blood pressure) and the lay term results in many irrelevant documents. Thus, the ATM is designed to increase recall at the cost of precision (refer to PubMed Help to understand how ATM works).

## Results

**Figure 3:**
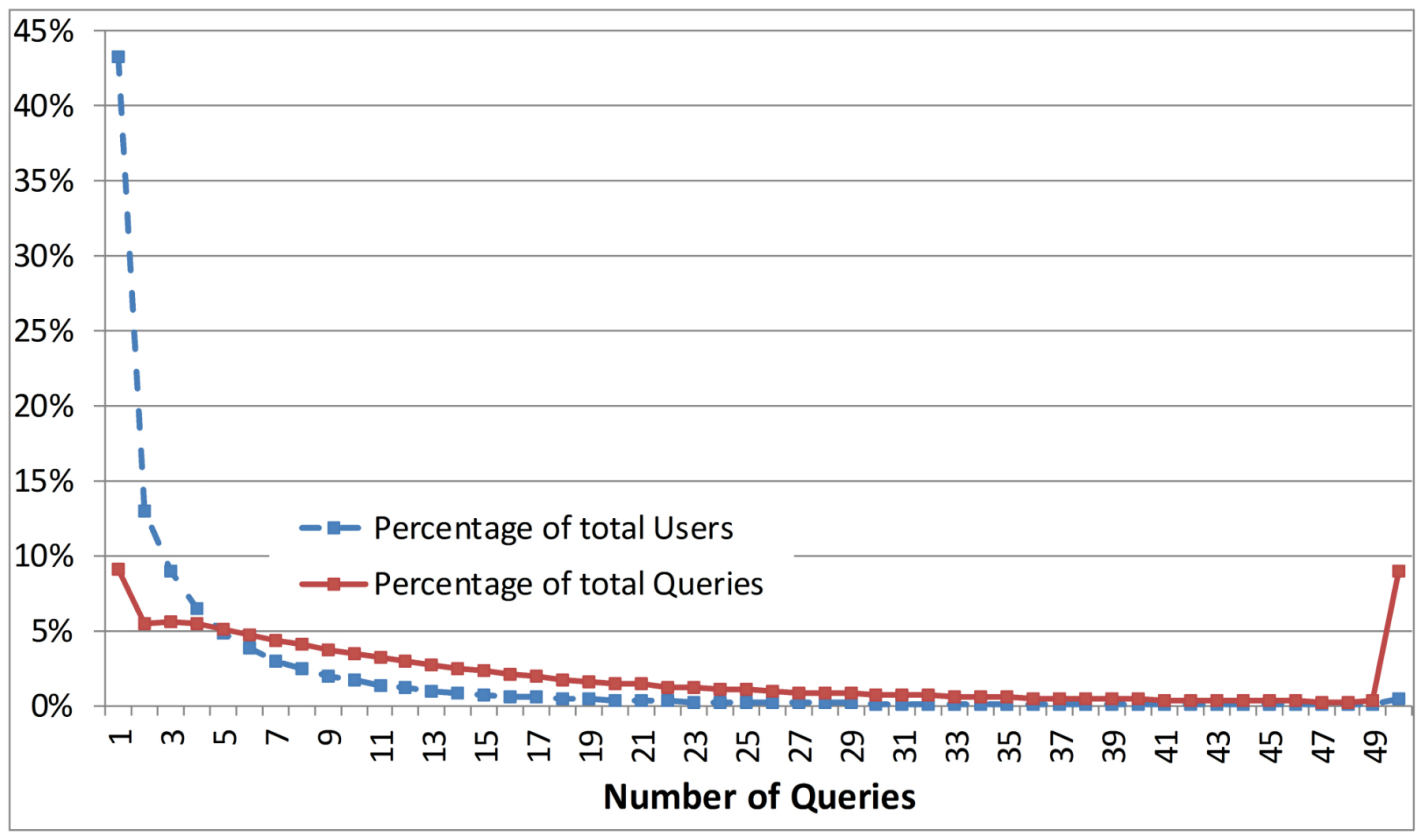
Percentage of users and queries per number of queries.

First, we performed some basic statistical analysis on query and session data. The number of queries per user ranged from 1 to 8544 (an extreme outlier) with an average of 4.77 queries per user (SD 15.11, median 2). Figure 3 presents the proportion of users that submitted different numbers of queries and the proportion of queries submitted by the corresponding users. Many PubMed users submitted one query. About two-fifths (43%) of users submitted one query that represented around 9% of the total queries. The rest of the users (57%) performed multiple queries and those queries represented about 90% of the total queries. More than half of PubMed users performed one or at most two queries for their information needs. There was a gradual decrement in the proportion of users as the number of queries increased.

**Figure 4:**
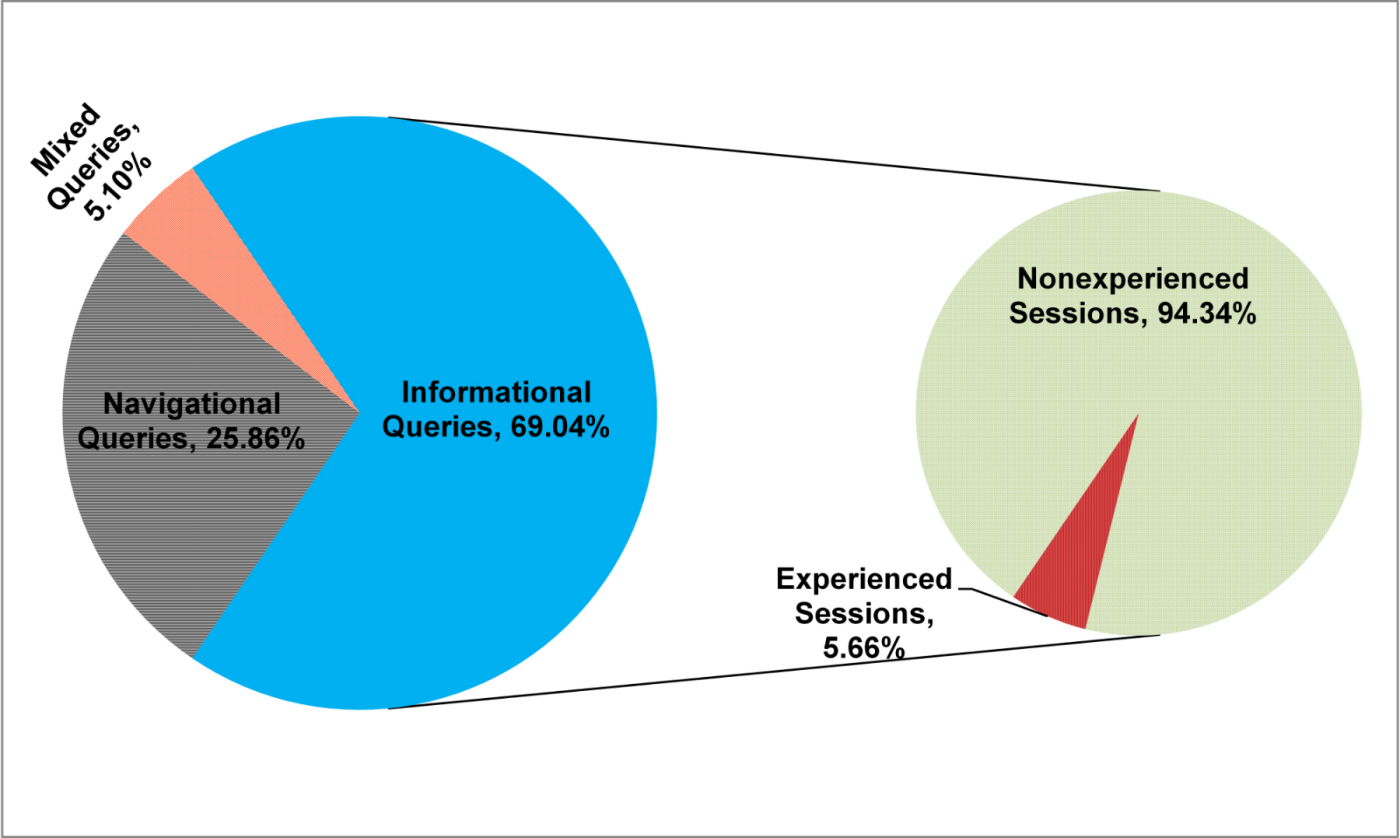
Query types and session types.

PubMed users may perform multiple IR sessions to fulfill their various information needs. In order to identify the purpose of each IR session, we categorized the queries in the log dataset as shown in Figure 2. Figure 4 presents the percentages of different query types. A total of 2,012,466 (69%) queries were identified as informational, 753,827 (26%) queries navigational, and 148,510 (5%) queries mixed. A total of 742,602 user sessions were identified from the informational queries. Because we compared experienced and nonexperienced search sessions, we further identified experienced and nonexperienced search sessions based on their system function usage from the user sessions (that are identified from the informational queries only, see Figure 4).

About 94% (=700,547/742,602) of the sessions were performed by nonexperienced-users and 6% (=42,055/742,602) of the sessions were performed by experienced users (see Figure 4). Some of the users (about 1.12%) performed both experienced and nonexperienced search sessions meaning that such sessions contain both experienced and nonexperienced queries. Since these users knew how to perform searches using advanced system functions, we considered them as experienced users. There are two possible explanations as to why they performed nonexperienced queries. First, they needed to express new concepts but there were no MeSH terms for the concepts. Thus, although they knew of advanced search functions such as MeSH terms, they could not avoid using natural language to describe concepts. Second, as Vibert et al (2009) [6] found, many PubMed users with search skills do not use search functions.

**Figure 5:**
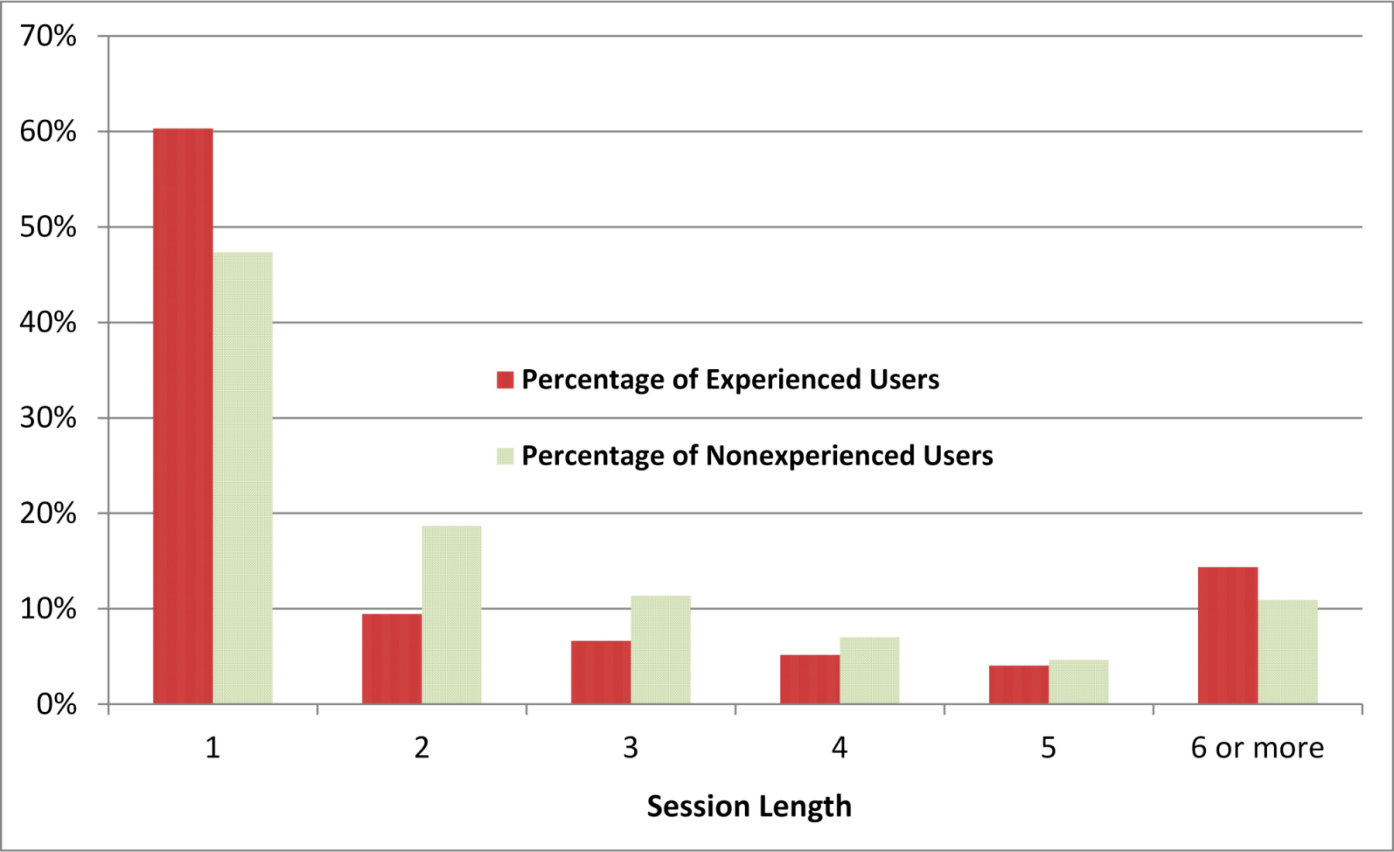
Percentages of experienced and nonexperienced users per session length (# of queries per session).

Figure 5 shows the histogram of the proportion of the experienced and nonexperienced users for the various session lengths (the number of queries in a session). Technically, the users in the figure indicate sessions. Because a user may have multiple sessions, a set of sessions that is performed by the same user cannot be matched with a specific (integer number of) session length, meaning that each session is independently treated in the analysis. For both of the groups, the proportion of users significantly decreased as the number of sessions increased. For experienced users, the session length ranged from 1 to 308 (an extreme outlier) with an average of 2.85 queries per session (SD 4.24, median 1). For nonexperienced users, session length ranged from 1 to 8522 (an extreme outlier) with an average of 2.7 (SD 11.61, median 2). As the standard deviation values indicate, session length variation of nonexperienced sessions was higher than that of experienced sessions. Figure 5 clearly shows the difference between experienced users and nonexperienced users in terms of session length. While for users whose session length was 1 (ie, an ideal IR), the percentage of experienced users was higher than that of nonexperienced users (25,365/42,055, 60.31% vs 331,337/700,547, 47.30%), for users whose session length was 2 or 3, the percentage of the experienced group was lower than that of the nonexperienced group. This session length difference indicates that experienced users completed their searches earlier than nonexperienced users.

**Figure 6:**
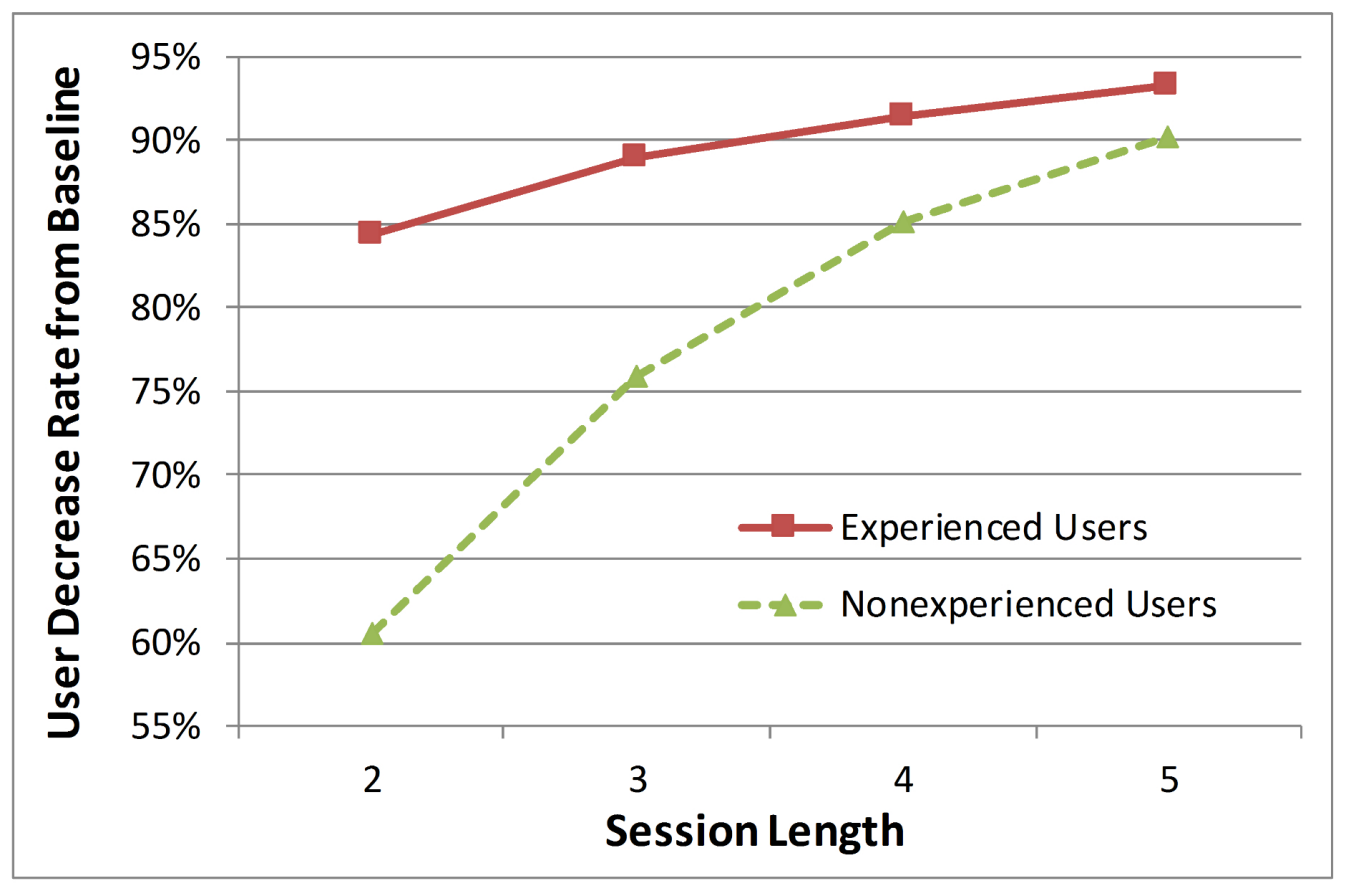
Decrease rates of experienced and nonexperienced users by session length (# of queries per session).

In addition, we measured user decrease rates of the experienced and nonexperienced users from the session length of 1 to 2, 3, 4, and 5. Because the ideal session length is 1 (meaning that a user fulfills his or her information need with only one query), the baseline session length should be 1 (the ideal session). Decrease rates from the baseline indicate the success of the IR session (at retrieving relevant documents). Figure 6 compares decrease rates from the baseline of the two user groups. The decrease rate of the experienced users at the session length of 2 was significantly higher than that of the nonexperienced group (the formula to calculate the rate of the experienced users at the session length of 2 is: 1 − # of experienced sessions at the session length of 2/# of experienced sessions at the session length of 1, or 1 – 3969/25,365 = 84.30%). The decrease rates of the two groups indicated that most experienced PubMed user sessions were closed within only one query (note the median of the session lengths was 1) (in other words, the initial or first query satisfied the users’ information needs) and nonexperienced user sessions (median of 2) were longer than those of the experienced group.

##  Discussion

### Principal Findings

In bibliographic searches like PubMed searches, procedural knowledge is an important factor to improve the overall performance of information retrieval. Procedural knowledge includes experience using online search systems and their search functions. Earlier studies demonstrated that PubMed users perform searches with higher recall and precision if PubMed search functions are used [25,26,85-89]. These studies used at most tens of human subjects for their experiments. In this study, to check the effect of IR functions on PubMed searches, we performed an analysis on a very large scale. The full-day PubMed log data we used contained nearly 3 million user queries issued by more than 0.6 million users. To our knowledge, this study is the first in the field of biomedical and health informatics to use log data containing nearly 3 million queries to compare search performance and behavior of experienced and nonexperienced users. For the analysis, we first categorized queries into informational or navigational based on their underlying intentions, and then identified 0.7 million informational query sessions from more than 2 million informational queries. An informational query session consisted of one or many informational queries in a row within a 20-minute session window. Sessions were further categorized into experienced and nonexperienced user sessions. To test our hypotheses, we compared experienced and nonexperienced users, and found that experienced PubMed users quickly retrieved relevant documents and nonexperienced PubMed users had longer search sessions than experienced users.

### Limitations

There are some limitations of this study. First, the PubMed query log data used in this study could have been biased in terms of IR function usage because the data contained search queries for one day only. Second, we used a predetermined time cutoff (20 minutes) for determining search sessions since the log data did not contain any session-related information. It is possible for a PubMed user to perform more than one session in 20 minutes. However, according to recent studies [6,84], most users complete their search session within 20 minutes. At the same time, it is not common that PubMed users spend more than 20 minutes on a search session; more than 65% of PubMed users perform one to three queries per session (see Figure 3). Third, the classification of users based on the use of search tags is not always correct. In other words, the user classification names (ie, experienced and nonexperienced user groups) do not always necessarily indicate that, for example, all the users in the nonexperienced user group are PubMed novice users. At the same time, we believe the group included some experienced users. There are two reasons why experienced users sometimes do not use search functions: first, in order to find “recently published” articles one must use natural language (nonMeSH terms) because those articles are not indexed yet (indexing lag); second, using MeSH terms requires one to search the MeSH database first before conducting PubMed searches (this is an additional step).

Fourth, we assumed if a session was closed within a few queries, the session was successful (meaning that their information needs were fulfilled) even if a session close does not always mean successful IR. This assumption is based on the fact that nearly 77% of users had only 1 to 3 queries in a session. We believe that most searches are successful. If most searches were unsuccessful, one would expect that most users would not use PubMed again. However, according to the NLM, the number of PubMed users has been increasing. In fact, there is no way to know if a session has been successful using the log data; using web log information is the only solution to this problem but this information is not available. We believe that some sessions that are closed within a few queries are unsuccessful. However, the gaps between the decrease rates of the experienced and nonexperienced users (especially at the session length of 2, see Figures 5 & 6) clearly indicate that most sessions that are closed within a few queries are successful. In fact, these limitations are related to the use of log data, rather than direct data from human subjects, for the analysis. In other words, the limitations are simply drawbacks of using log data that we cannot readily overcome.

### Current Applicability of the Log Data Analysis to PubMed

It is unknown when the PubMed query data were collected, for confidentiality reasons. However, they are at least 9 years old. One might argue that this study based on old log data is still currently applicable, because the NLM has added many features to improve the performance and user interface of PubMed. Some examples are related citations, automatic term mapping, and PubMed Clinical Queries. PubMed is significantly different from how it was 9 years ago, in terms of the user interface and internal processes for better information retrieval. However, it is imperative to ascertain whether the new features and user interface retrieve documents that are more relevant or lead to better PubMed searches. Studies have found that most PubMed users *still* have difficulty finding relevant documents for patient care in PubMed and do not want to use PubMed for their information needs (instead they want to use UpToDate and/or Google).

There are many recent studies (published in 2010 or later) that found that physicians prefer UpToDate and/or Google to PubMed, and that UpToDate and/or Google provide more answers to clinical questions. Thiele and colleagues (2010) [90] evaluated four search tools (Google, Ovid, PubMed, and UpToDate) widely used to answer clinical questions. They found that Google was the most frequently used search engine for patient care, and Google and UpToDate were faster and brought more clinical answers than PubMed and Ovid. Shariff and colleagues (2013) [91] compared the performance of searches in PubMed and Google Scholar by evaluating the recall and precision of the searches (the first 40 search result records were analyzed) to determine how well search engines answered nephrological questions. The recall of Google Scholar was two times higher than that of PubMed (indicating documents twice as relevant) while the precision of Google Scholar was slightly higher than that of PubMed (indicating less irrelevant documents in the search result). Another advantage of Google Scholar was that it provided nearly three times more links to full-text documents than PubMed. Duran-Nelson and colleagues (2013) [92] carried out a survey to uncover how internal medicine residents use resources (such as UpToDate, Google/Google Scholar, and PubMed) for point-of-care (POC) clinical decision making. The top two resources the residents used daily at the POC were UpToDate and Google. Of interest, although the residents thought both UpToDate and PubMed provided trustworthy information for patient care, only 20 residents used PubMed daily while nearly 140 residents used UpToDate daily. In addition, the biggest barrier to using PubMed was speed (it took more time to find clinical answers with PubMed). Cook and colleagues (2013) [93] performed a study similar to Duran-Nelson’s (Duran-Nelson et al, 2013) [92]. This focus group study (based on a brief survey) showed that physicians used UpToDate two times as much as PubMed, and physicians regarded PubMed as less useful in POC learning due to the time required to find relevant information through PubMed searches. Sayyah Ensan and colleagues (2011) [94] compared PubMed Clinical Queries and UpToDate to determine their ability to answer clinical questions and the time required to find answers. Their findings were that (a) physicians obtain more answers using UpToDate (76%) than PubMed Clinical Queries (43%), and (b) the median times spent retrieving answers using UpToDate and PubMed Clinical Queries were 17 minutes and 29 minutes, respectively. Nourbakhsh and colleagues (2012) [95] evaluated PubMed and Google Scholar with four clinical questions. The first 20 citations/results were analyzed and classified into three relevance groups (clearly relevant, possibly relevant, and not relevant). They found Google Scholar retrieved more relevant documents than PubMed (80% vs 67.6%). Thiele and colleagues (2010) [96] conducted a survey of medical students, residents, and attending physicians on computer use and four search engines widely used to answer clinical questions (Google, Ovid, PubMed, and UpToDate), and compared the search engines in terms of accuracy, speed, and user confidence. Results showed that 33% and 32% of physicians used UpToDate and Google, respectively, for answering their clinical questions, while only 13% of physicians used PubMed. The authors found that Google and UpToDate answered more clinical questions correctly and more quickly than PubMed.

In sum, the findings of these recent studies indicate that the information retrieval features of PubMed are inferior to other electronic resources or search engines such as UpToDate and Google. In other words, most PubMed users still have considerable difficulty obtaining relevant documents/information despite its many new features. As a result, physicians spend more time finding relevant information with PubMed. This problem is critical for PubMed because recent studies still show that the main barrier to POC learning is lack of time [90] [91] [92] [93] [97] [98]. We believe, based on these recent studies that virtually nothing has changed in terms of information-seeking behavior and PubMed from the user’s perspective.

### Conclusions

The PubMed log analysis indicated that experienced PubMed users quickly retrieved relevant documents in terms of session length and nonexperienced PubMed users had longer search sessions than experienced users. We believe there are a few potential solutions to this problem. First, the NLM could design and provide a novel PubMed user interface for nonexperienced users so that they can readily utilize advanced search functions without special training in PubMed. Second, because it is imperative for health professionals (especially physicians) to learn the system functions and MeSH vocabulary for better PubMed searches, the NLM could award grant funding only to institutes that regularly train health professionals in PubMed search skills. Third, the NLM could develop a sophisticated relevance-sorting algorithm similar to Google’s, so that PubMed users can quickly find relevant documents. Currently, PubMed provides a relevance sorting option. However, it is not the default sorting option as of 17 June 2015 and we believe there should be a significant improvement to the sorting algorithm. This PubMed search problem is not just an information retrieval issue but also a health care practice matter, because health professionals, especially physicians, could significantly improve the quality of patient care and effectively educate chronic patients using clinical and medical information and knowledge obtained from PubMed searches.

## Abbreviations

ATM: Automatic Term Mapping
IR: information retrieval
MeSH: Medical Subject Heading
NLM: National Library of Medicine
